# A Low-Molecular-Weight BDNF Mimetic, Dipeptide GSB-214, Prevents Memory Impairment in Rat Models of Alzheimer’s Disease

**DOI:** 10.32607/actanaturae.11755

**Published:** 2022

**Authors:** P. Yu. Povarnina, A. A. Volkova, O. N. Vorontsova, A. A. Kamensky, T. A. Gudasheva, S. B. Seredenin

**Affiliations:** Research Zakusov Institute of Pharmacology, Moscow, 125315 Russia; Lomonosov Moscow State University, Faculty of Biology, Moscow, 119991 Russia

**Keywords:** brain-derived neurotrophic factor, dimeric dipeptide mimetic, Alzheimer’s disease, scopolamine, streptozotocin, memory

## Abstract

Brain-derived neurotrophic factor (BDNF) is known to be involved in the pathogenesis of Alzheimer’s disease (AD). However, the pharmacological use of full-length neurotrophin is limited, because of its macromolecular protein nature. A dimeric dipeptide mimetic of the BDNF loop 1, bis-(N-monosuccinyl-L-methionyl-L-serine) heptamethylene diamide (GSB-214), was designed at the Zakusov Research Institute of Pharmacology. GSB-214 activates TrkB, PI3K/AKT, and PLC-γ1 in vitro. GSB-214 exhibited a neuroprotective activity during middle cerebral artery occlusion in rats when administered intraperitoneally (i.p.) at a dose of 0.1 mg/kg and improved memory in the novel object recognition test (0.1 and 1.0 mg/kg, i.p.). In the present study, we investigated the effects of GSB-214 on memory in the scopolamine- and steptozotocin-induced AD models, with reference to activation of TrkB receptors. AD was modeled in rats using a chronic i.p. scopolamine injection or a single streptozotocin injection into the cerebral ventricles. GSB-214 was administered within 10 days after the exposure to scopolamine at doses of 0.05, 0.1, and 1 mg/kg (i.p.) or within 14 days after the exposure to streptozotocin at a dose of 0.1 mg/kg (i.p.). The effect of the dipeptide was evaluated in the novel object recognition test; K252A, a selective inhibitor of tyrosine kinase receptors, was used to reveal a dependence between the mnemotropic action and Trk receptors. GSB-214 at doses of 0.05 and 0.1 mg/kg statistically significantly prevented scopolamine-induced long-term memory impairment, while not affecting short-term memory. In the streptozotocin-induced model, GSB-214 completely eliminated the impairment of short-term memory. No mnemotropic effect of GSB-214 was registered when Trk receptors were inhibited by K252A.

## INTRODUCTION

Alzheimer’s disease (AD) is the most common cause of dementia, accounting for 60–80% of all dementia cases, while no effective pathogenetic therapy exists today for this disease [1].

Over the past two decades, regulation of the activity of neurotrophin receptors, and the brain-derived neurotrophic factor (BDNF) in particular, has been viewed as a new strategy for treating neurodegenerative diseases. BDNF maintains neuronal viability and synaptic plasticity, playing an important role in the processes of learning and memory. Data indicative of BDNF involvement in the pathogenesis of AD have been published [2, 3, 4]. Reduced BDNF expression is already observed at the early stage of the disease and correlates with an accumulation of β-amyloid and the hyperphosphorylated tau protein [5]. The favorable effects of exogenous BDNF have been demonstrated in various AD models. BDNF ensures neuronal protection under conditions of β-amyloid toxicity both in vitro and in vivo [6]. Insertion of the BDNF gene within a lentiviral vector into J20 transgenic mice (carrying mutations in the gene encoding the amyloid precursor protein) prevented the death of the cells of the entorhinal cortex and improved cognitive functions [7]. It has been shown using another genetic model of AD (P301L mice carrying the mutant tau protein gene) that stable human BDNF gene expression restored the BDNF level, thus preventing neuronal and synaptic degeneration in the hippocampus, as well as cognitive disorders [8]. However, the gene therapy has such shortcomings as invasiveness, high cost, and the risk of adverse effects related to the pleiotropic effect of BDNF.

The clinical use of BDNF is impeded by its poor penetration through the blood–brain barrier and rapid degradation [9]. Low-molecular-weight BDNF mimetics with improved pharmacokinetic properties are currently being developed [10, 11]. Activity of the low-molecular-weight BDNF mimetic 7,8-dihydroxyflavone, a TrkB receptor agonist, was determined using AD models [12, 13, 14].

**Fig. 1:**
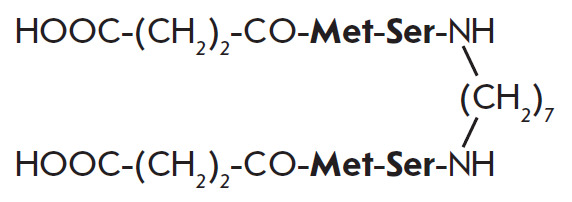
The dimeric dipeptide mimetic of the BDNF loop 1 GSB-214

A dimeric dipeptide mimetic of the BDNF loop 1, GSB-214 (bis-(N-monosuccinyl-L-methionyl- L-serine) heptamethylene diamide), was designed and synthesized at the Zakusov Research Institute of Pharmacology based on the hypothesis that the most exposed domains of the loop-like neurotrophin structures (most frequently, the central domains of their β turns) exhibit pharmacophoric properties [15] [RU Patent 2410392, 2011; US Patent 9683014 B2, 2017; CN Patent 102365294 B, 2016; EU Patent 2397488, 2019; IN Patent 296506, 2018] (Fig. 1).

Earlier, Western blotting showed that incubation of HT-22 mouse hippocampal cells in the presence of GSB-214 for 5–180 min results in the activation of TrkB receptors and the conjugated PI3K/Akt and PLC-γ1 signaling pathways, but not the MAPK/ERK signaling pathway [10]. It has been shown using HT-22 cells that GSB-214 at micro-nanomolar concentrations exhibits neuroprotective activity under oxidative stress [15].

The dipeptide GSB-214 (administered i.p. at doses of 0.1–0.5 mg/kg) exhibited in vivo neuroprotective activity in a rat model of transient middle cerebral artery occlusion [16] and antidiabetic activity in a streptozotocin-induced model of diabetes in mice [17]. Taking into account the findings regarding the similarity of the pathogenesis of diabetes and AD [18], the antidiabetic properties of GSB-214, along with the neuroprotective properties, indicate that there is promise in studying the effects of the dipeptide in AD models.

The objective of our work was to investigate the effect of GSB-214 on memory in the scopolamine-and streptozotocin-induced models of AD, as well as evaluate its mnemotropic activity as a function of the activation of Trk receptors.

## EXPERIMENTAL

**Materials**

The dipeptide GSB-214 was synthesized at the Medicinal Chemistry Department of the Zakusov Research Institute of Pharmacology according to the procedure described earlier [14]; 96% chromatographic purity (HPLC), [α]^25^_D_ = +9.0° (0.4 in DMF), T_melt_ = 162–163°C. Scopolamine (Acros Organics, USA), streptozotocin, and K252A (Sigma Aldrich, USA) were used.

**Animals**

The experiments were conducted using male Wistar rats (weight, 230–260 g) procured from the Andreevka Branch of the Research Center for Biomedical Technologies, the Federal Medical- Biological Agency (FMBA). The animals were kept in a vivarium with ad libitum feeding and access to water and natural light–dark cycle. The behavioral experiments were carried out at a time interval between 10 a.m. and 2 p.m. (local time). The animal experiments were carried out in compliance with international regulations (Directive 2010/63/EU of the European Parliament and of the Council of the European Union of September 22, 2010, on the protection of animals used for scientific purposes). The experiments were approved by the Biomedical Ethics Committee of the Zakusov Research Institute of Pharmacology (Protocol No. 3 dated February 18, 2021).

**Scopolamine-induced model of AD**

The rats were randomly assigned to the following groups: Control (n = 9), Scopolamine (SC) (n = 10), SC + GSB-214 (0.05 mg/kg) (n = 10), SC + GSB-214 (0.1 mg/kg) (n = 9), and SC + GSB-214 (1.0 mg/kg) (n = 10). Scopolamine in normal saline was injected i.p. to rats at a dose of 2 mg/kg during 20 days. GSB-214 in distilled water was injected i.p. at doses of 0.05, 0.1, and 1.0 mg/kg during 10 days after exposure to scopolamine. The rats in the Control group received equiva lent volumes of normal saline, instead of scopolamine, and distilled water, instead of GSB-214, according to the same scheme. The rats in the SC group received scopolamine and distilled water.

The novel object recognition test was carried out on days 32–33.

**Fig. 2:**
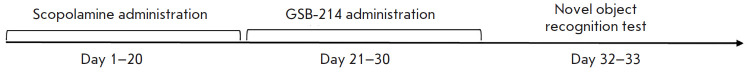
The scheme of the experiment on the mnemotropic effects of GSB-214 in the scopolamine-induced AD model

The scheme of the experiment is shown in Fig. 2.

**Streptozotocin-induced model of AD**

The rats were randomly assigned to the following groups: Control (n = 10), Streptozotocin (STZ) (n = 7), and STZ + GSB-214 (0.1 mg/kg) (n = 8). STZ in citrate buffer was stereotactically injected into the cerebral ventricles at a dose of 3 mg/kg (AP = −1.0; L = 1.5; depth, 3.5). The injection volume was 3 µL per ventricle; the injection rate was 1 µL/min. One hour after the exposure, the rats received an i.p. injection of GSB-214 (0.1 mg/kg) and, then, received injections once daily during 13 days. The rats in the Control group were injected with equivalent volumes of citrate buffer, instead of STZ, and distilled water, instead of GSB-214, according to the same scheme. The rats in the STZ group received STZ and distilled water.

**Fig. 3:**
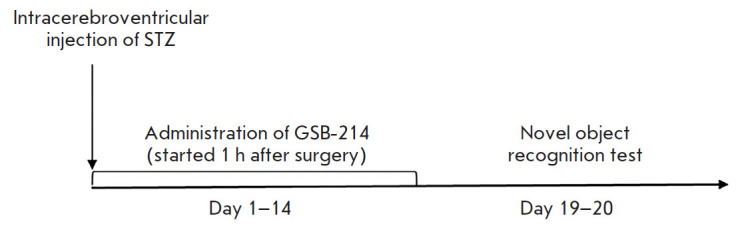
The scheme of the experiment on the mnemotropic effects of GSB-214 in the streptozotocin-induced AD model. STZ – Streptozotocin

The novel object recognition test was carried out on days 19–20. The scheme of the experiment is shown in Fig. 3.

**The novel object recognition test**

This test is based on the natural rodents’ instinct to investigate novel objects [19]. It is widely used for assessing both short-term and long-term memory [20].

The test was conducted in T4 cages identical to the home cages where the animals had been housed throughout the study. A rat was first placed into an empty cage with the floor covered with sawdust for 4 min to adapt.

The familiarization phase. Two identical objects not familiar to the rat were placed in the two nearest corners of the cage. The time spent exploring the objects was recorded during 4 min. The rat was then returned to its home cage.

Test. A new pair of objects was placed in the same corners of the cage; one object was identical to those presented to the rats during the familiarization phase, while the other was unfamiliar. The time spent exploring the familiar and novel objects was recorded during 4 min. The test was carried out 1 h (test 1) and 24 h (test 2) after the familiarization phase to record the short-term and long-term memory, respectively. Different unfamiliar objects were used in test 1 and test 2. Exploration was defined as sniffing, with the distance between the animal•s snout and the object being ≤ 2 cm.

The discrimination index was used as the memory criterion [21]; it was calculated using the formula: DI = (T_novel_ – T_fam_)/(T_novel_ + T_fam_), where Tnovel was the time spent exploring a novel object and T_fam_ was the time spent exploring a familiar object. The KD values > 0 meant that the animal remembered the object presented to it at the familiarization phase.

**Pharmacological inhibitory analysis**

The rats were randomly assigned to the following groups: Control (distilled water and 1% DMSO in normal saline, n = 12), GSB-214 0.1 mg/kg (GSB-214 and 1% DMSO, n = 13), GSB-214 0.1 mg/kg + K252A 100 μg/kg (n = 12), and K252A 100 μg/kg (distilled water and K252A, n = 13). GSB-214 at a dose of 0.1 mg/kg or an equivalent amount of distilled water was administered i.p. 20 min after the i.p. injection of K252A (100 μg/kg) in 1% DMSO or 1% DMSO. The novel object recognition test was started after 24 h. The dose of GSB-214 was chosen based on earlier experiments [22].

**Statistical analysis**

Statistical analysis of the experimental data was performed using the GraphPad Prism 8.0 software (GraphPad Software, USA). The statistical significance of differences in the discrimination index was assessed using one-way ANOVA, followed by pairwise intergroup comparisons using the Dunnett’s test or two-factor ANOVA followed by pairwise intergroup comparisons using the Tukey’s test.

The data were presented as the mean ± standard error of the mean. Differences were considered statistically significant at p < 0.05.

## RESULTS

**The dipeptide GSB-214 prevents long-term memory impairment in the scopolamine-induced model of AD**

Compared to the control group, chronic administration of scopolamine significantly reduced the discrimination index in both test 1 (1 h after becoming familiar with the objects, p = 0.0212) and test 2 (24 h after becoming familiar with the objects, p = 0.0077), thus indicating that short-term and long-term memory, respectively, was impaired (Table 1). Chronic administration of GSB-214 at doses of 0.05 and 0.1 mg/kg prevented long-term memory impairment (p = 0.0177 and 0.0304 vs. SC group, respectively), although it had no effect on short-term memory. No activity was observed for the dipeptide GSB-214 when administered at a dose of 1.0 mg/kg (Table 1).

**Table 1:** The effects of GSB-214 in the scopolamine-induced model of amnesia in the novel object recognition test

Group	Number of animals per group	Discrimination index	
		Test 1 (1 h)	Test 2 (24 h)
Control	9	0.57 ± 0.05	0.53 ± 0.06
SC	10	0.3 ± 0.06^*^	0.23 ± 0.06^**^
SC+GSB-214 (0.05 mg/kg)	10	0.48 ± 0.07	0.48 ± 0.04^#^
SC+GSB-214 (0.1 mg/kg)	9	0.45 ± 0.07	0.47 ± 0.05#
SC+GSB-214 (1.0 mg/kg)	10	0.33 ± 0.06	0.44 ± 0.08

The data are presented as the mean ± standard error of the mean. ^**^p < 0.01, ^*^p < 0.05 compared to the Control group; ^#^p < 0.05 compared to the SC group (one-way ANOVA, the Dunnett’s test).

**The dipeptide GSB-214 prevents short-term memory impairment in a streptozotocin-induced model of AD**

In the streptozotocin-induced model of AD, we uncovered significant memory impairment in the rats in the STZ group 1 h after becoming familiar with the objects (p = 0.0045), but not after 24 h (Table 2). Therefore, in this experimentally induced model of AD, rats experienced short-term, rather than long-term, memory impairment, which is typical of the early stage of the disease [23]. GSB-214 at a dose of 0.1 mg/kg yielded a statistically significant correction of this impairment (p = 0.0032); the discrimination index in the group of animals receiving treatment was 4.8-fold higher compared to that in the STZ group (Table 2).

Hence, the dipeptide GSB-214 completely inhibited short-term memory impairment in the streptozotocin-induced model of AD.

**Table 2:** The effects of GSB-214 on short-term memory in the novel object recognition test for the streptozotocin- induced model of AD

Group	Number of animals per group	Discrimination index	
		Test 1 (1 h)	Test 2 (24 h)
Control	10	0.46 ± 0.07	0.49 ± 0.05
STZ	7	0.1 ± 0.08^**^	0.43 ± 0.07
STZ+GSB-214 (0.1 mg/kg)	8	0.48 ± 0.07^##^	0.48 ± 0.03

The data are presented as the mean ± standard error of the mean. ^**^p < 0.01 compared to the Control group; ^##^p < 0.01 compared to the STZ group (one-way ANOVA, the Dunnett’s test).

**The mnemotropic activity of GSB-214 depends on the activation of Trk receptors**

In order to confirm the involvement of the activation of Trk receptors in the mnemotropic effects of GSB-214, we studied how K252A, an inhibitor of these receptors, influences the effects of GSB-214 in the novel object recognition test. Table 3 shows that the dipeptide GSB-214 significantly improved long-term memory as the discrimination index in the test after 24 h in this case increased approximately 1.5- fold compared to that in the control group. This effect was completely eliminated by injecting a K252A inhibitor 20 min before the exposure to GSB-214. K252A per se did not affect the rats’ memory. The studied compounds were found to exhibit no effect on the short-term memory of the rats (test 1) (Table 3).

**Table 3:** The Trk receptor inhibitor completely eliminates the mnemotropic effect of GSB-214 on long-term memory

Group	Number of animals per group	Discrimination index	
		Test 1 (1 h)	Test 2 (24 h)
Control	12	0.53 ± 0.07	0.47 ± 0.06
GSB-214 (0.1 mg/kg)	13	0.5 ± 0.05	0.73 ± 0.03^***^
GSB-214 (0.1 mg/kg)^+^ K252A	12	0.53 ± 0.06	0.36 ± 0.03^####^
K252A	13	0.54 ± 0.06	0.43 ± 0.05

The data are presented as the mean ± standard error of the mean. ^***^p < 0.001 compared to the Control group; ^####^p < 0.0001 compared to the GSB-214 group (twoway ANOVA, the Tukey’s test).

## DISCUSSION

Earlier, we had found that a single-dose BDNF dipeptide mimetic GSB-214 administered i.p. (0.1 and 1.0 mg/kg) had a favorable effect on the long-term memory of rats in the novel object recognition test [22].

In this study, we investigated the mnemotropic activity of GSB-214 in the same test in the scopolamine-and streptozotocin-induced models of AD.

The scopolamine-induced amnesia model is commonly used for evaluating potential therapeutic agents for treating AD [24, 25, 26]. Chronic exposure to scopolamine causes cholinergic deficit that is mainly induced by blockade of acetylcholine receptors and, therefore, cognitive impairment [25]. In our modification of the model [24], the impairment induced by chronic exposure to scopolamine and its subsequent discontinuation (see the scheme of the experiment in Fig. 2) is attributed to the activation of feedback mechanisms, which first increase the density and affinity of acetylcholine receptors and subsequently induce the cholinergic deficit due to accelerated binding of the “available” acetylcholine.

The model of AD induced by intracerebroventricular injection of streptozotocin is also com monly used, has been validated, and studied well [27, 28]. Streptozotocin, a diabetogenic toxin, enters cells by binding to glucose transporter 2, because it is structurally similar to a sucrose molecule [28]. Intracerebral administration of streptozotocin induces insulin resistance and impairs brain glucose metabolism [29]. It causes neuropathological symptoms typical of AD, such as accumulation of β-amyloid and hyperphosphorylated tau protein, oxidative stress, as well as neuronal and synaptic death [30, 31, 32, 33]. Like the scopolamine-induced model of AD, the streptozotocin-induced model is associated with memory disorders [31, 33].

We have revealed short-term and long-term memory impairment in the scopolamine-induced model of AD, which is consistent with the published data [26, 34]. The dipeptide GSB-214 eliminated only long-term memory impairment, while having no effect on short-term memory. This finding agrees with our earlier data obtained under physiological conditions in the novel object recognition test [22]. We assume that the revealed effect of GSB-214 can be attributed to the activation of the PI3K/Akt post-receptor signaling pathway, which was demonstrated earlier in in vitro experiments [10]. Serine/threonine protein kinase mTOR, one of the major protein synthesis regulators, is a component of the PI3K/Akt pathway [35]; it is viewed as the key factor in memory consolidation and, therefore, long-term memory formation [36]. It was found, using the novel object recognition test, that mTOR inhibition impairs long-term memory, but not short-term memory , in rats [37]. A hypothesis can be put forward that the effects of GSB-214 in the scopolamine-induced model of AD are related to the improvement of memory consolidation via the activation of the TrkB/PI3K/Akt/mTOR signaling pathway. We have demonstrated by pharmacological inhibitory analysis that the mnemotropic activity of GSB-214 is caused by an activation of the Trk neurotrophin receptors with which the PI3K/Akt/mTOR signaling pathway is associated.

In the streptozotocin-induced model, we observed only short-term memory impairment, which can be indicative of relatively mild neurodegenerative changes being characteristic of early AD [38]. GSB-214 eliminated this impairment. Since no effect of GSB-214 on short-term memory under physiological conditions was observed previously [22], it is fair to assume that memory was recovered due to the increase in neuronal viability under the exposure to streptozotocin-induced toxicity. The neuroprotective effects of GSB-214 were revealed earlier in in vitro experiments [15], as well as in a rat model of ischemic stroke induced by transient middle cerebral artery oc clusion [16]. These effects, like the mnemotropic ones, are presumably associated with the activation of the PI3K/Akt signaling pathway. This pathway is known to mediate neuroprotection by inhibiting pro-apoptotic proteins and increasing the expression of anti-apoptotic proteins [39]. PI3K/Akt was shown to mediate a reduction of the activity of glycogen synthase kinase 3β (GSK-3β), which is involved in increased β-amyloid production and hyperphosphorylation of the tau protein [40].

Interestingly, the previously revealed antidiabetic activity of GSB-214 proved dependent on the activation of the PI3K/Akt pathway, as shown by a pharmacological inhibitory analysis [17]. Since it is well-known that AD and diabetes mellitus have a similar pathogenesis [18], this fact supports the idea that the PI3K/Akt pathway also contributes to the effects of GSB-214 in a streptozotocin-induced model reproducing all the major pathophysiological mechanisms of AD.

**Fig. 4:**
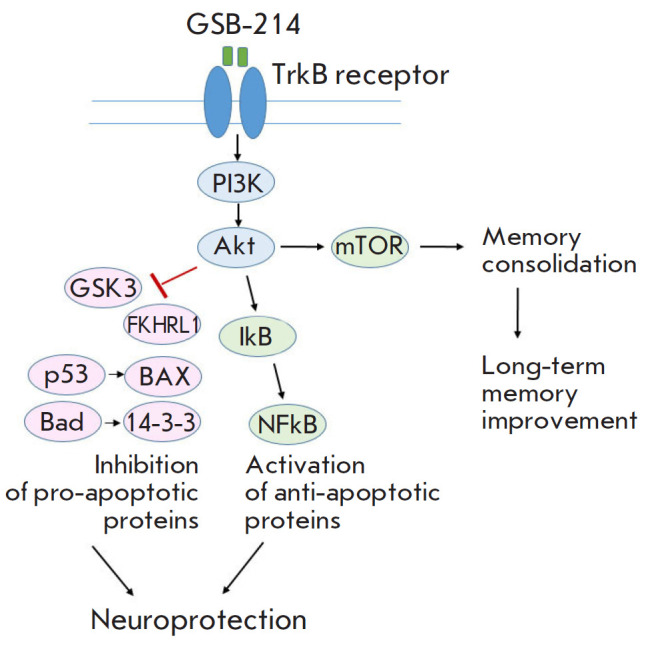
The putative mechanisms of action of the BDNF dipeptide mimetic GSB-106 in AD models

Figure 4 shows the putative mechanisms of action of GSB-214 in AD models. Additional studies are needed to identify the exact mechanisms of action of GSB-214 in an experimentally induced model of AD.

Activation of the PI3K/AKT signaling pathway by the dipeptide GSB-214, which had previously been identified in in vitro experiments [10], may promote neuroprotection by inhibiting pro-apoptotic proteins and activating anti-apoptotic proteins, as well as improve memory consolidation and, therefore, long-term memory through the activation of the regulator of mTOR protein synthesis.

## CONCLUSIONS

Therefore, the low-molecular-weight BDNF mimetic GSB-214 dipeptide eliminates induced memory impairment in rats in the scopolamine- and streptozotocin-induced models of Alzheimer’s disease. The effect of GSB-214 depends on the activation of Trk receptors.

## Abbreviations

BDNF: brain-derived neurotrophic factor
SC: scopolamine
STZ: streptozotocin
AD: Alzheimer’s disease

## References

[1] (2019). 2019 Alzheimer’s disease facts and figures. Alzheimer’s Dementia..

[2] Giuffrida M.L., Copani A., Rizzarelli E. (2018). Aging (Albany. NY)..

[3] Iulita M.F., Bistué Millón M.B., Pentz R., Aguilar L.F., Do Carmo S., Allard S., Michalski B., Wilson E.N., Ducatenzeiler A., Bruno M.A. (2017). Neurobiol. Dis..

[4] Amidfar M., de Oliveira J., Kucharska E., Budni J., Kim Y.K. (2020). Life Sci..

[5] Wang Z.H., Xiang J., Liu X., Yu S.P., Manfredsson F.P., Sandoval I.M., Wu S., Wang J.Z., Ye K. (2019). Cell Rep..

[6] Arancibia S., Silhol M., Moulière F., Meffre J., Höllinger I., Maurice T., Tapia-Arancibia L. (2008). Neurobiol. Dis..

[7] Nagahara A.H., Mateling M., Kovacs I., Wang L., Eggert S., Rockenstein E., Koo E.H., Masliah E., Tuszynski M.H. (2013). J. Neurosci..

[8] Jiao S.S., Shen L.L., Zhu C., Bu X.L., Liu Y.H., Liu C.H., Yao X.Q., Zhang L.L., Zhou H.D., Walker D.G. (2016). Transl. Psychiatry..

[9] Kopec B., Zhao L., Rosa-Molinar E., Siahaan T. (2020). Med. Res. Arch..

[10] Gudasheva T.A., Povarnina P.Y., Tarasiuk A.V., Seredenin S.B. (2021). Med. Res. Rev..

[11] Longo F.M., Massa S.M. (2013). Nat. Rev. Drug Discov..

[12] Zhang Z., Liu X., Schroeder J.P., Chan C.-B., Song M., Yu S.P., Weinshenker D., Ye K. (2014). Neuropsychopharmacology..

[13] Aytan N., Choi J.K., Carreras I., Crabtree L., Nguyen B., Lehar M., Blusztajn J.K., Jenkins B.G., Dedeoglu A. (2018). Eur. J. Pharmacol..

[14] Bollen E., Vanmierlo T., Akkerman S., Wouters C., Steinbusch H.M.W., Prickaerts J. (2013). Behav. Brain Res..

[15] Gudasheva T.A., Tarasyuk A.V., Pomogaibo S.V., Logvinov I.O., Povarnina P.Yu., Antipova T.A., Seredenin S.B. (2012). Russ. J. Bioorganic Chem..

[16] Gudasheva T.A., Povarnina P., Logvinov I.O., Antipova T.A., Seredenin S.B. (2016). Drug Des. Devel. Ther..

[17] Yagubova S.S., Ostrovskaya R.U., Gudasheva T.A., Seredenin S.B. (2020). Bull. Exp. Biol. Med..

[18] de la Monte S.M., Wands J.R. (2008). J. Diabetes Sci. Technol..

[19] Ennaceur A., Delacour J. (1988). Behav. Brain Res..

[20] Antunes M., Biala G. (2012). Cogn. Process..

[21] Beldjoud H., Barsegyan A., Roozendaal B. (2015). Front. Behav. Neurosci..

[22] Volkova A.A., Povarnina P.Yu., Nikiforov D.M., Gudasheva T.A., Seredenin S.B. (2022). Pharm. Chem. J..

[23] Richter N., Beckers N., Onur O.A., Dietlein M., Tittgemeyer M., Kracht L., Neumaier B., Fink G.R., Kukolja J. (2018). Brain..

[24] Ostrovskaya R.U., Mirzoev T.Kh., Firova F.A. (2001). Experimental and Clinical Pharmacology..

[25] van Dam D., De Deyn P.P. (2006). Nat. Rev. Drug Discov..

[26] Bhuvanendran S., Kumari Y., Othman I., Shaikh M.F. (2018). Front. Pharmacol..

[27] Rai S., Kamat P.K., Nath C., Shukla R. (2013). J. Neuroimmunol..

[28] Kamat P.K., Kalani A., Rai S., Tota S.K., Kumar A., Ahmad A.S. (2016). Mol. Neurobiol..

[29] Kamat P.K. (2015). Neural Regen. Res..

[30] Salkovic-Petrisic M., Hoyer S. (2007). J. Neural Transm. Suppl..

[31] Ravelli K.G., Rosário B. dos A., Camarini R., Hernandes M.S., Britto L.R. (2017). Neurotox. Res..

[32] Bassani T.B., Turnes J.M., Moura E.L.R., Bonato J.M., Cóppola-Segovia V., Zanata S.M., Oliveira R.M.M.W., Vital M.A.B.F. (2017). Behav. Brain Res..

[33] Afshar S., Shahidi S., Rohani A.H., Komaki A., Asl S.S. (2018). Psychopharmacol..

[34] Mugwagwa A.T., Gadaga L.L., Pote W., Tagwireyi D. (2015). J. Neurodegener. Dis..

[35] Switon K., Kotulska K., Janusz-Kaminska A., Zmorzynska J., Jaworski J. (2017). Neuroscience..

[36] Hernandez P.J., Abel T. (2008). Neurobiol. Learn Mem..

[37] Jobim P.F.C., Pedroso T.R., Werenicz A., Christoff R.R., Maurmann N., Reolon G.K., Schröder N., Roesler R. (2012). Behav. Brain Res..

[38] Porsteinsson A.P., Isaacson R.S., Knox S., Sabbagh M.N., Rubino I. (2021). J. Prev. Alzheimer’s Dis..

[39] Reichardt L.F. (2006). Philos. Trans. R. Soc. B Biol. Sci..

[40] Long H.Z., Cheng Y., Zhou Z.W., Luo H.Y., Wen D.D., Gao L.C. Front..

